# Supplementary material for: Multiple sclerosis and COVID-19: A retrospective study in Iran
Source: PLoS One. 2023 Mar 23;18(3):e0283538. doi: 10.1371/journal.pone.0283538 (PMC10035930; doi:10.1371/journal.pone.0283538)
Supplement: Attachment — Submitted filename: response to reviewers.docx [file pone.0283538.s001.docx]

When submitting your revision, we need you to address these additional requirements.

1. Please ensure that your manuscript meets PLOS ONE's style requirements, including those for file naming. The PLOS ONE style templates can be found at 
<https://journals.plos.org/plosone/s/file?id=wjVg/PLOSOne_formatting_sample_main_body.pdf> and 
<https://journals.plos.org/plosone/s/file?id=ba62/PLOSOne_formatting_sample_title_authors_affiliations.pdf>

Thank you for your note. The manuscript is based on the formatting guidelines.

2. We note that you have indicated that data from this study are available upon request. PLOS only allows data to be available upon request if there are legal or ethical restrictions on sharing data publicly. For more information on unacceptable data access restrictions, please see <http://journals.plos.org/plosone/s/data-availability#loc-unacceptable-data-access-restrictions>.

a) If there are ethical or legal restrictions on sharing a de-identified data set, please explain them in detail (e.g., data contain potentially sensitive information, data are owned by a third-party organization, etc.) and who has imposed them (e.g., an ethics committee). Please also provide contact information for a data access committee, ethics committee, or other institutional body to which data requests may be sent.

b) If there are no restrictions, please upload the minimal anonymized data set necessary to replicate your study findings as either Supporting Information files or to a stable, public repository and provide us with the relevant URLs, DOIs, or accession numbers. For a list of acceptable repositories, please see <http://journals.plos.org/plosone/s/data-availability#loc-recommended-repositories>.

We will update your Data Availability statement on your behalf to reflect the information you provide.

We will upload data as Supporting Information files.

3. Your ethics statement should only appear in the Methods section of your manuscript. If your ethics statement is written in any section besides the Methods, please delete it from any other section.

It was corrected in the manuscript.

Review Comments to the Author

Reviewer #1:

First, the grammar is very poor. The entire manuscript needs a significant revision for grammar.

Thank you for your note. We revised the manuscript for English language and grammar.

Regarding the content, there are several major problems.

1. What does COVID infection mean? Does it mean symptomatic + PCR confirmation? Does it mean antigen testing? Is it patient reported or physician confirmed?

The diagnosis was based on the physician's judgment, which used lab tests, including PCR and clinical signs and symptoms.

2. Employed vs. unemployed needs to be further explored. At a condition that associated with COVID, were employed people working in a private office or in a crowded market with exposure to many other people? Were unemployed people confined to their homes or moving around their urban centers?

Employed means having a job working for a company or another person with usual exposure to other people, while unemployed means not having a permanent job or being a university student and also may have usual exposure to other people.

We didn’t feel the need for further exploration with our patients since they were strict about self-protection and self-isolation due to their underlying disease. Conversely, our patients were completely informed on the protocols for preventing the contraction of COVID infection since the beginning of the pandemic.

3. What is "addiction"? Addiction to what - smoking?

In this study, we asked the patients about their lifestyle habits, such as tobacco, alcohol, and substance abuse (opioid, heroin, methamphetamine, or other stimulant substance). We added details in the methods section.

4. What is "physical disease"?

In this study, we only recruited subjects without any important chronic diseases, such as diabetes, hypertension, cardiovascular, cerebrovascular, and respiratory diseases, and "physical disease" means complications accompanying MS other than the conditions noted above.

5. What medications were these patients using for their MS?

The patient's medications were added in table 1.

6. Other risk factors for COVID include diabetes, hypertension, lung disease, etc - did these patients have those?

The patients who had chronic non-communicable diseases (diabetes, hypertension, cardiovascular, cerebrovascular, and respiratory diseases) and those who did not agree to participate in the study were excluded.

Reviewer #2:

 Sedighi et al present a retrospective evaluation of CoVID-19 related outcomes in patients with MS in a referral clinic located in Kerman, Iran. The authors assessed factors associated with COVID-19 infection, along with the effectiveness and untoward effects of the CoVID-19 vaccine. It is helpful to get a report on the impact of CoVID19 on the Iranian MS population. There are several points that should be addressed in the article:
1. The references for the global prevalence of MS are not current and should be updated. The most recent data puts the world MS prevalence estimate at 2.8 million based on the 3rd edition of the Atlas of MS (Walton C, et al. Mult Scler. 2020 Dec; 26(14): 1816–1821. doi: 10.1177/1352458520970841).

Thank you for your note. We updated the MS prevalence.

2. How representative is the 367-study sample of the Kerman MS clinic population? How many patients were excluded either due to failure to obtain consent or for other reasons? Was this a convenience population based on lab testing or clinic follow-ups? These issues should be documented and explained.

We used the registry of MS patients who received care from the only referral hospital in Kerman province. Out of 548 registered cases, we recruited 367 (66.9 %); those who did not agree or had a major non-communicable disease such as diabetes, hypertension, cardiovascular disease, cerebrovascular disease, and respiratory disease were excluded. We added details in the methods section.

3. How was SARS-CoV2 infection confirmed in the study population and over what period of time? It is difficult to judge the 35% incidence of “getting CoVID-19” in the study population if the details are not presented. Was the diagnosis made in laboratory-confirmed patients based on either polymerase chain reaction or serology tests vs. presumptive diagnosis based on symptoms or exposures? Also, the time period of tracking infections is important with the arrival of various CoVID-19 variants which have different infectivity rates. These data should be documented in the methods.

The patients with COVID-19 in this study were those who had the disease since the beginning of the pandemic that includes various CoVID-19 variants and the diagnosis was based on physician judgment, which used lab tests including PCR and clinical signs and symptoms. We added details in the methods section.

4. The MS disability status (EDSS) is obtained on only 57/367 study participants. This is missed opportunity to assess how neurological disability impacts the study outcomes. In several studies in other countries, neurological disability made COVID-19 infection more severe and more likely to be recognized. On a related note, I’m wondering if the MS population at the clinic was more severely impaired neurologically than comparable MS populations with such a high (69%) unemployment rate in a young adult population.

Based on the information in table 1, EDSS was obtained from all the patients, of whom 309 were categorized as mild and 57 as moderate to severe.

The EDSS provides a total score ranging from 0 to 10: 0-2.5 refers to people with a mild degree of disability, 3-5 refers to people with a moderate degree of disability, and 5.5-10 refers to severe disability.

We must add that the majority of the samples are women and unemployment is not just because of their MS conditions, a considerable proportion of healthy young adult women or university students do not have full-time jobs, so the unemployment rate in our sample is high.

5. Were MS disease modifying therapies documented in the study population? If not, why not. Patients with MS taking CD20 disease modifying therapies (e.g., rituximab and ocrelizumab) have been shown to be at increased risk for CoVID-19 and have less robust responses to vaccines.

**Table**. Type of DMT and getting COVID- 19 in each group

|  | Covid-19 | |  |
| --- | --- | --- | --- |
| DMT | **Yes** | **No** | **P-value** |
| Injectable | 49 | 86 | 0.14 |
| Anti CD20 | 23 | 40 |  |
| S1P receptor modulator (Fingolimod) | 20 | 22 |  |
| Natalizumab | 2 | 16 |  |
| Oral (Dimethyl fumarate, Teriflunomide) | 23 | 48 |  |
| None | 11 | 25 |  |

In response to the comment, we assessed and recorded the history of patients including disease modifying therapies. In summary, and incompatible with our expectation, we did not find any significant difference in contracting covid-19 between users and non-users of these drugs (table above), however, some finding was also reported in some references in the following.

Based on the above explanation, we added the following sentences in the result section.

We compared the prevalence of COVID-19 in patients with MS based on the type of DMT, our result showed that there is not a significant difference in the prevalence of COVID-19 according to their medications.

1. Bsteh G, Assar H, Hegen H, Heschl B, Leutmezer F, Di Pauli F, Gradl C, Traxler G, Zulehner G, Rommer P, Wipfler P. AUT-MuSC investigators. COVID-19 severity and mortality in multiple sclerosis are not associated with immunotherapy: Insights from a nation-wide Austrian registry. PLoS One. 2021;16(7): e0255316.

2. Louapre C, Collongues N, Stankoff B, Giannesini C, Papeix C, Bensa C, Deschamps R, Créange A, Wahab A, Pelletier J, Heinzlef O. Clinical characteristics and outcomes in patients with coronavirus disease 2019 and multiple sclerosis. JAMA neurology. 2020 Sep 1;77(9):1079-88.

6. Significant digits after the decimal point for odds ratios and confidence intervals should be limited to 1 or 2 (e.g., 1.1 or 1.11).

 It was corrected in the manuscript.

7. Clear labeling of the risk factor of interest should be presented in the abstract in results as opposed to the generic variable label. Male sex vs. sex and current employment vs employment status.

 It was corrected in the abstract section.
