# Supplementary material for: Multiple sclerosis and COVID-19: A retrospective study in Iran
Source: PLoS One. 2023 Mar 23;18(3):e0283538. doi: 10.1371/journal.pone.0283538 (PMC10035930; doi:10.1371/journal.pone.0283538)
Supplement: Attachment — Submitted filename: response to reviewers2.docx [file pone.0283538.s002.docx]

Reviewer #2:

While many of the comments were addressed in the revision, there are still issues that remain in the updated manuscript. The authors should address the following:

1. Significant grammatical errors remain in the revised article making several sections difficult to read.

Thank you for your note. We revised the manuscript for English grammar.

2. In the abstract, it would be more informative to include quantitative numerical descriptions rather than qualitative statements. For example, in the abstract it states that Kerman is “one of the largest cities in Iran.” It would be helpful if the abstract mentioned the numerical population of the city. Instead of stating “most of the patient with multiple sclerosis in this study received at least one dose of the vaccine,” it would be helpful to state what percentage of the patients in the study received the vaccine.

It was revised in the abstract section.

3. In the first paragraph of the results section, it would be helpful to list the mean EDSS score of the patient and explain how the patients’ disease activity was stratified into mild, moderate or severe based on the EDSS score.

It was revised in the method and result section.

4. The table in the results section references "physical disease,” but this term is not further explained in the results or the methods sections. Without further explanation of this term earlier in the paper, the reader will have difficulty understanding what this term refers to.

 It was corrected in the manuscript.

5. In the results section, it states that there was no difference in the prevalence of COVID-19 between the different DMT groups. It would be helpful to mention in the discussion section whether Anti CD20 agents were included. If anti-CD 20s were not included, it may be helpful to discuss that this may have impacted the data as other studies have demonstrated that Anti-CD use impacted vaccine efficacy.

It was added in the discussion.

6. Table 5 lists the number of adverse effects, but the types of adverse effects were never defined. It would be beneficial to list in the table the different types of adverse effects that were included.

It was added in the table.
