# Supplementary material for: Multiple sclerosis and COVID-19: A retrospective study in Iran
Source: PLoS One. 2023 Mar 23;18(3):e0283538. doi: 10.1371/journal.pone.0283538 (PMC10035930; doi:10.1371/journal.pone.0283538)
Supplement: Attachment — Submitted filename: response3.docx [file pone.0283538.s003.docx]

Journal Requirements:

Please review your reference list to ensure that it is complete and correct. If you have cited papers that have been retracted, please include the rationale for doing so in the manuscript text, or remove these references and replace them with relevant current references. Any changes to the reference list should be mentioned in the rebuttal letter that accompanies your revised manuscript. If you need to cite a retracted article, indicate the article’s retracted status in the References list and also include a citation and full reference for the retraction notice.

Thank you for your note. The reference list has been checked and corrected.

Additional Editor Comments:

Results:

Line 134-136 please re-write the paragraph as listed below:

Furthermore, we compared the prevalence of COVID-19 in patients with MS based on the type of DMT, and our results showed no significant difference in the prevalence of COVID-19 based on the kind of DMT taken by the patients.

It was corrected in the manuscript.

Keep R as upper case for reference throughout the tables. This needs to be done for all tables.

Discussion:

Line 177-179: Please add a little discussion here about the effects of CD 20 and COVID-19 severity in your patient population and how it affected the vaccine efficacy in your patient population?
 It was added in the discussion.

Conclusion:

Line 197-199 Please re-write paragraph as listed below:

We found that the prevalence of COVID-19 in the patients with MS was higher than in the other studies, which could be an alarm for patients with chronic and immunosuppressive diseases in the healthcare system in Iran.

It was corrected in the manuscript.
